# Factors Associated with Suicide Attempts and Suicides in the General Population of Andalusia (Spain)

**DOI:** 10.3390/ijerph16224496

**Published:** 2019-11-14

**Authors:** Yolanda Mejías-Martín, Juan de Dios Luna del Castillo, Candela Rodríguez-Mejías, Celia Martí-García, Juan Pablo Valencia-Quintero, María Paz García-Caro

**Affiliations:** 1 Department of Mental Health, General University Hospital Virgen de las Nieves, 18014 Granada, Spain; yolanda.mejias.sspa@juntadeandalucia.es; 2 Biostatistics Unit, Faculty of Medicine, University of Granada, 18016 Granada, Spain; jdluna@ugr.es; 3 Department of Intensive Care, General University Hospital Virgen de las Nieves, 18014 Granada, Spain; candelarm@correo.ugr.es (C.R.-M.); juanpablovalenciaq@yahoo.es (J.P.V.-Q.); 4 Department of Nursing, Faculty of Health Sciences, University of Malaga, 29071 Malaga, Spain; 5 Department of Nursing, Faculty of Health Sciences, University of Granada, 18016 Granada, Spain; mpazgc@ugr.es

**Keywords:** suicide, suicide attempt, population groups, emergency medical services

## Abstract

Discrepant results have been published by studies comparing deaths by suicide with attempted suicides. This study aimed to determine factors associated with suicides and attempted suicides in Andalusia (Spain) between 2007 and 2013, comparing sex, age, year, and suicide method between these populations. A retrospective study was conducted of data on deaths by suicide and attempted suicides over a seven-year period, calculating the sex and age rates for each behavior. Adjusted Poisson regression was used to analyze the association with study variables, and incidence rate ratios were estimated. During the seven-year study period, 20,254 attempted suicides and 5202 deaths by suicide were recorded. The prevalence of attempted suicide did not differ between the sexes, whereas the prevalence of deaths by suicide was three-fold higher among males than among females and increased with higher age. The most frequently used method was the same in males and females for suicide attempts, but differed between the sexes for suicides. The combined influence of sex and age was greater in the model for death by suicide than in the model for attempted suicide. The key differentiating factor was the method used, while the finding of greatest concern was the suicide behavior among the elderly. Preventive strategies should take these differences into account.

## 1. Introduction

The World Health Organization [1] considers suicide to be a major public health problem whose prevention requires the intervention of multiple disciplines and approaches. It is a complex behavior involving personal, social, psychological, biological, cultural, and environmental factors [2].

There is broad but not complete consensus in the scientific literature on the events that constitute to suicidal behavior [3,4,5,6,7]. It is described by the WHO [2] as a process that involves multiple behaviors, including ideation, planning, attempt, and suicide. In this study, we differentiate between deadly suicidal behavior, i.e., actions that cause death, designated “deaths by suicide”, and non-deadly suicidal behavior, i.e., suicidal actions that do not cause death, designated “attempted suicides” [8]. The lethality of the selected method, among other circumstances, defines whether an attempt is in fact suicidal behavior, which is a complex concept [9,10,11,12,13].

Other authors have proposed that people who attempt suicide and those who actually die by suicide are different populations with distinct characteristics [14,15,16], although there would be a degree of overlap depending on the variables considered [17,18,19,20]. Comparisons between these populations (attempted suicides vs. suicides) have reported some differences in age, sex, method [10,13,18,21,22] and/or clinical variables [9,17,19].

A history of suicide attempts is known to increase the risk of suicide [2,23,24,25,26], although many suicide victims have not made any previous attempt [9,13,18,19].

Discrepant results have been published by studies on attempted and completed suicides, and comparisons are hampered by differences in sample sizes (generally small) and study populations, recruited in hospital emergency departments, psychiatry departments, and/or primary care offices in different geographic localizations. Inconsistencies in findings may also be attributable to the lack of an internationally accepted protocol for recording data on suicide attempts. In fact, only limited data are available on attempts, based on self-reports in wider community surveys and on descriptions of self-inflicted lesions in the records of hospital emergency departments and other health centers [2,27]. However, it seems that the majority of people who die by suicide or attempt suicide have no previous contact with a mental health center or hospital [7,18], which was observed in only one-third of suicide victims in one study [28]. Likewise, suicide attempts do not always lead to admission to a hospital emergency department [29,30,31], with one study finding that only one-quarter of attempts ended in the emergency department of a public hospital [32]. There has been a call for improvements in research on suicidal behavior, with the adoption of fresh approaches [3,33]. In the present study, we considered suicide and attempted suicide to be carried out by two different populations, collecting data on attempts from the records of pre-hospital care services as a reliable source of information on suicide attempts in general populations [31] and data on suicides from the National Statistics Institute (Instituto Nacional de Estadística (INE)) [34]. Information on any differences in characteristics between these populations could be of major value for designing specific preventive programs [35].

The objectives of this study were to determine factors associated with death by suicide and attempted suicide in the general population of Andalusia (Spain) between 2007 and 2013 and to compare sex, age, year, and method between suicide attempts and deaths by suicide.

## 2. Methods

We conducted an observational, cross-sectional, retrospective, and comparative study of data on deaths by suicide and attempted suicides in Andalusia (Spain) during the period between 1 January 2007 and 31 December 2013.

Andalusia is the largest region of Spain, with a population of more than eight million [36]. The pre-hospital emergency service (Empresa Pública de Emergencias Sanitarias (EPES)) of the Andalusia public health system responds to emergency phone calls throughout the region. Calls to the EPES are resolved by telephone, by an emergency team in situ, or by evacuating the affected individual to the hospital emergency department. All call related data are stored in the EPES Information System (SIEPES).

Data on suicide attempts were obtained from SIEPES records, selecting codes that define an attempted suicide as previously reported by our group (see [31]), i.e., 305 (305.4, 305.8), E950 to E959, and E980 to E989 in CIE9, and X84 in CIE10, adding E969 because of its connection with X84 or the aforementioned CIE9 codes in the SIEPES database. The International classification of diseases (ICD-9 and ICD-10) codes were both used in the database, and analogies between them were considered. These criteria excluded potential suicide attempts that were not labeled as X84, did not lead to resource mobilization, and were not labeled as suicide attempt by the healthcare team. Cases of individuals under 15 years of age were excluded from the study due to the very small number of cases

Data on deaths by suicide between 2007 and 2013 were gathered from the INE.

### 2.1. Study Variables

Data were gathered for both deaths by suicide and attempted suicides on the province, the year, the sex and age of the individual, and the method used. Predictor variables were age, sex, and the suicide method, and the models were adjusted for province and year. Cases were classified into eight self-harm groups according to ICD-9/ICD-10 codes, including X84 (Table 1).

Variables available from the INE on deaths from suicide included sex, age, province, and year, although only the sex of individuals was available for the suicide method used. With respect to the EPES data, information on the attempted suicide method was missing in 60% of cases; therefore, the results of their analysis should be treated with caution.

### 2.2. Statistical Analysis

Given reports by various authors [37,38,39] on the influence of age, sex, and geographic localization (in the present case, province) on differences between suicide and attempted suicide behaviors, multivariate analysis was conducted for each behavior, adjusting for these variables.

Crude suicide and attempted suicide rates were calculated by year, sex, and age. Multivariate Poisson regression was used to compute the incidence rate ratio (IRR) for attempted suicides and for suicides, adjusting the IRR in each model for year, province, age, and sex. In a second step, the interaction of age and sex was assessed, using the likelihood ratio test, and was found to be significant; therefore, IRRs for age were estimated by sex, with the corresponding CI. A multivariate Poisson regression model could not be used to compute the IRR for the suicide method, because this was recorded in only 40% of the suicide attempts. For this reason, a model was developed for each method by sex. The large number of missing data on age prevented the development of a similar model for age. In all cases, the Poisson goodness-of-fit test was applied. STATA 14.1 (StataCorp LP., College Station, TX, USA) was used for statistical analyses.

### 2.3. Ethics

All data were treated anonymously in accordance with national legislation on Personal Data Protection (Law 15/1999). The study complied with the principles of the 2008 revision of the Helsinki Declaration of 1975 and was approved by the Ethics Committee of the EPES.

## 3. Results

Between 1 January 2007 and 31 December 2013, 20,254 suicide attempts (excluding cases in which death occurred before arrival of the emergency team) and 5202 suicides were recorded.

### 3.1. Rates and Ratios of Suicide and Attempted Suicide

As shown in Table 2, a larger number of attempts was not associated with more suicides. The attempted suicide rate was similar between males and females, but the suicide rate was three-fold higher in males. The ratio of suicide and attempted suicide was 9.58 among females vs. 2.32 among males.

Suicide and attempted suicide rates did not follow a monotonic pattern over the seven-year study period (Table 2). The highest suicide rate was in 2008 (12.0); the highest attempted suicide rate was in 2013 (46.5); and the highest suicide to attempted suicide ratio was in 2011 (4.39).

Analysis by age showed that the highest attempted suicide rate was in the 40–44 year age group (60.8/100,000 inhabitants), while the highest suicide rate was in the age interval between 80 and 84 years (25.3/100,000 inhabitants). There was a progressive increase in suicide rates with older age, whereas there was an increase in attempted suicide rates up to the age of 40–44 years, followed by a progressive decrease up to the age of 75–79 years and an increase from the age of 80 years onwards. The lowest ratio of suicide to attempted suicide was between the ages of 75 and 84 years, being 0.90 at 75–79 years and 0.98 at 80–84 years, whereas the highest ratio (8.03) was between the ages of 15 and 29 years.

**Figure 1:**
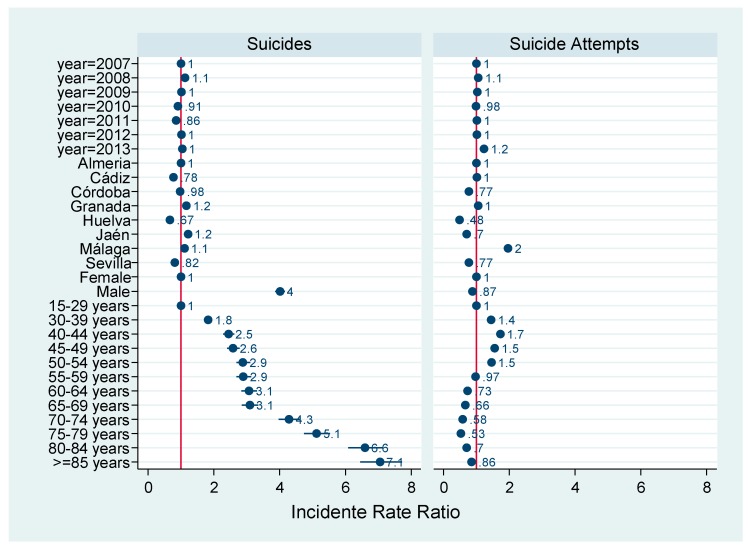
Incidence rate ratios for suicides and attempted suicides.

Figure 1 depicts associations observed between suicides and attempted suicides by year, sex, and age. Among the eight provinces of the region of Andalusia, the province with the highest attempted suicide rate was Malaga (2.0), whereas the highest suicide rates were in Granada and Jaen (1.2 for each)

### 3.2. Association between Suicide and Attempted Suicide by Year, Sex, and Age

The results of Poisson regression analyses of these data are listed in Table 3.

The adjusted IRR for suicide attempts was 0.12-fold lower in males than in females, while the incidence of suicides was 4.02-fold higher in males (95% confidence interval (CI) = 3.76; 4.29). There was little interannual variation in IRR for suicides except in 2010 and 2011, when it was 0.09- and 0.14-fold lower, respectively, than in 2007. However, there was an increase in the adjusted IRR for attempted suicides over the seven-year period, with peaks in 2009 (1.11) and 2013 (1.22).

Table 4 exhibits the results of the adjusted model for the relationship of age with suicides and attempted suicides for each sex. For suicides, the sex–age interaction was significant in all cases (males and females). For attempted suicides, the sex–age interaction was significant in all cases with the exception of the 55–59-year-old age group in both sexes and the over-80-year-old group in males.

### 3.3. Association between Suicide and Attempted Suicide According to the Method Used

The method was recorded by the medical team in only 40% of the attempted suicide cases (*n* = 6306), preventing repetition of the statistical analysis conducted for the global sample.

As shown in Table 5, the most frequent method used for attempted suicide was drug poisoning for both sexes, whereas the most frequent methods for suicide were hanging for males (86.8%) and drug poisoning (45%) or drowning (40%) for females.

All methods were used in a lesser proportion by the females than by the males for both suicides and attempted suicides with the exception of drug poisoning, which was more often used for attempted suicide by females (IRR of 1.47). The least frequently used method for either attempted suicide or suicide was by firearm, which was also the method least likely to be used by males for either behavior (IRR of 0.04 and 0.34, respectively).

## 4. Discussion

This study in Southern Spain revealed differences between adults (>15 years) who attempted suicide and died by suicide in our setting over a seven-year period. There was little inter-annual variation in either attempted suicide or suicide rates, and no correlation was observed between these behaviors.

Given that our database of attempted suicides would not include those that did not produce a call to the EPES, the attempted suicide rate and therefore the ratio of suicide attempts to deaths by suicide were likely underestimated. However, this bias would not affect the comparison between IRRs obtained from adjusted Poisson models for suicide attempts and suicides, which was the main objective of our study. We therefore believe that this bias would have little influence on our main findings. It is also possible that the suicide database may include patients who did not resort to the EPES during the study period, which would reflect differences between people who die by suicide and those who attempt suicide, as demonstrated in our comparison of the adjusted model for each population.

### 4.1. Sex Differences between Suicide Attempts and Deaths by Suicide.

The results for suicides and attempted suicides differed between males and females, as reported in previous studies, which have described the “gender paradox of suicidal behavior” [40,41]. Thus, it was found that males more frequently die by suicide than females, except in China and some rural areas of India [26,42], while females more frequently attempt suicide, although the gender difference is smaller than for suicide.

However, we observed only a minimal difference between males and females in attempted suicide rates. Freeman et al. [43] reported variations in the gender balance for attempted suicides among four European countries, with major differences being observed in Hungary, Ireland, and Portugal, but no significant difference in Germany. The U.S. National Institute of Mental Health [44] also described a minimal difference in the prevalence of attempted suicide between males and females, whereas a study in England found that females were more likely to report an attempted suicide in comparison to males [45]. Data from other regions of Spain [19] have shown a much wider gender gap. These discrepancies may be attributable to differences in methodology, type of study population, and the definition of attempted suicide [5,46].

In our study, the rates of death by suicide were lower than reported in other European regions [39] for both males and females. A higher male:female ratio (4.02) was found than in the other European regions (3.1), being similar to the mean ratio reported for Europe by the WHO [2].

### 4.2. Age and Sex Specific Differences between Attempted Suicides and Suicides

In our study, the influence of age differed between suicides and attempted suicides by sex. Thus, attempted suicide was less frequent among females than males at younger ages and more frequent among females at higher ages until ≥70 years, when it was similar between the sexes. Our results for the younger age group contrast with the finding in North America that more than three-quarters of attempted suicides during adolescence were by females [47] and the report in England that the highest prevalence among females was in the 16–24 year age group [45]. The prevalence of attempted suicide by males was highest among those aged 30–44 years and >75 years in our study, whereas it was also highest among those aged 25–44 years in the United Kingdom [48], but was minimal among the elderly. Our findings in males contrast markedly with data from the U.S. [49], where suicide attempts were most frequent between the ages of 18 and 25 years and those by over 50-year-olds represented only 0.1 % of the total.

Deaths by suicide were more prevalent among individuals aged 70 years or more in our study, whereas rates were found to be highest in the 45–59 year age group in the U.S. [44] and the U.K. [48]. However, considering males alone, the frequency of suicides was highest among the elderly in the U.S. [44,50], although it remained highest among 45–59-year-olds in the U.K. [48].

### 4.3. The Method Used Exerts an Influence on Suicide and Attempted Suicide

The suicide method and its lethality are generally considered to be key differential factors between attempted suicide and suicide [21,35,39,51,52,53]. It has been observed that males commonly choose more lethal methods in comparison to females [21,24,35,39,51,52]. Our results are consistent with this observation, although all methods were more frequently used by the males than females for suicides and also for attempted suicides with the exception of drug poisoning, the predominant method used by females to attempt suicide. It has previously been proposed that the same method tends to be more lethal when used by males than by females for death by suicide or attempted suicide [35,39,52]. Unlike in populations from North America [54] or Australia [35], firearms were infrequently used to attempt or die by suicide in our population, which may be related to differences in national firearm regulations [55]. The main suicide method for the males in our study was hanging, as observed in other European [39,56] and Spanish [19,37] regions, and drug poisoning was the main method for the females, similar to findings in the USA and different European populations [39,52,57].

### 4.4. Vulnerability of Elderly Men and Women to Suicidal Behavior

Among age groups, suicide was especially prevalent in the over 70-year-olds of both sexes. A study in Canada observed that the presence of physical diseases was associated with a reduced risk of suicide in both sexes at all ages, but the presence of psychiatric disease was associated with an increased suicide risk, especially among women aged between 70 and 84 years [58]. However, an investigation in Australia found that the highest risk of suicide was among elderly men with comorbidities [59]. These data suggest the need for health care organizations to consider the presence of physical diseases in males [60] and psychiatric disorders in females [61] as risk factors for suicide among the elderly.

Studies in elderly populations have reported that suicide attempts are often more lethal than in young people because they are more likely to plan their suicide and more often live alone [54,62,63]. Suicide or attempted suicide by the elderly has also been associated with poor social integration and self-perception as a burden on others [64]. According to other researchers, suicide may be seen as a solution to personal and social decline or may even be related to a resistance to seeking help among men, due to the masculine stereotype of emotional inhibition [65,66,67,68]. Some authors have attributed a higher susceptibility in some elderly males to the possible loss of “chromosome Y” in peripheral blood cells [69]. Further research in elderly populations is required to elucidate these issues, using primary care and care home data.

It is therefore possible to consider different risk population profiles for suicide attempts and deaths by suicide, which can be useful in the design of strategies to prevent one behavior or the other.

From the perspective of preventing these behaviors, an important contribution of these findings, obtained in a general population, is that the model for the effects of age and sex on attempted suicide is very different from the model for their effects on death by suicide. The models depict two different risk populations and therefore two distinct target groups for possible preventive measures. In the case of suicide attempts, this would include individuals between the ages of 30 and 50 years, for whom the risk is 1.4- to 1.7-fold higher overall, being slightly higher among the women than among the men (1 / 0.87 = 1.15). In the case of deaths by suicide, the targeting of measures should take account of the four-fold higher risk for men than for women and the increased risk between the ages of 40 and 69 years, with a marked rise after the age of 70 years. These distinct profiles should be considered in the design of specific preventive and surveillance measures in primary care and community centers and care homes, among others.

### 4.5. Strengths and Limitations

Analysis of separate adjusted models for suicides and attempted suicides reinforced our findings on differences between these phenomena. The population based perspective is a major strength of this study, which for the first time compares the differential effects on suicides and suicide attempts of conventionally considered factors.

Our findings on differences between attempted suicide and suicide rates are of limited value because they are based on global figures rather than individual cases, preventing estimations of the number of attempts made by individuals over the study period. However, despite this limitation in the variables available for analysis, the results of our adjusted models for attempted suicide and suicides demonstrated some relevant differences between these behaviors.

A further limitation is that we could not identify suicide attempts made by the same individual over the seven-year period or determine whether individuals dying by suicide had made previous suicide attempts; therefore, the same individual could be assigned to both groups or included multiple times in the attempted suicide group. However, despite the likelihood that this would have reduced between-group differences, we obtained statistically significant findings, suggesting that some of these effects may even be under-represented in our study.

Finally, the large amount of missing data on the method used to attempt suicide may have produced an important bias in the results for this variable, although it is not possible to know whether it would have produced an overestimation or an underestimation.

## 5. Conclusions

In this study population, the influence of sex differed between suicides and attempted suicides. The impact of age also varied between suicides and attempted suicides, finding a greater risk of the former with older age, while its influence on suicide attempts was weaker and less consistent. Sex and age both proved highly influential in the explanatory model for suicide, while they had a lesser effect in the model for attempted suicide due to factors that could not be evaluated with the present study design.

The means used for attempted suicide or suicide differed between the two behaviors, and its influence differed between the sexes. All methods were more frequently utilized by the men with the exception of drug poisoning, the commonest method selected by the women for attempted suicide, although it was more frequently used by the men for suicide. The present results indicate differences in risk profile between people who die by suicide and those who attempt this act, suggesting that distinct preventive strategies may be warranted against these behaviors.

The main study weakness was the large amount of missing information in the database on the methods used to attempt suicide. The WHO has called for the collection of data on suicide attempts to be standardized, and emergency care professionals need to be aware of the importance of recording information on the method used, which can be a useful indicator of the intentionality of the act.

Further research is needed on factors that might explain gender differences in the method used to attempt or die by suicide and also, given our worrying findings, on suicidal behavior among the elderly.

## Figures and Tables

**Table 1:** Codes and descriptions of methods, with equivalence of ICD-10 and ICD-9 codes.

Method	Description	ICD-10	ICD-9
Poisoning by drugs	Self-inflicted poisoning with psychotropic drugs, narcotics, or other drugs; or non-dependent substance abuse	X60, X61, X62, X63, X64	E950, E980, 305 (305.4, 305.8), 969
Poisoning by other means	Intentional self-inflicted poisoning by exposure to alcohol, gases, chemical products, etc.	X65, X66, X67, X68, X69	E951, E952, E981, E982
Hanging	Intentional self-inflicted harm by hanging, strangulation, suffocation, including cases when its accidental or intentional nature is unknown	X70	E953, E983
Drowning	Intentional self-inflicted harm by drowning and submersion, including cases when its accidental or intentional nature is unknown	X71	E954, E984
Firearms	Intentional self-inflicted harm by firearm, including cases when its accidental or intentional nature is unknown	X72, X73, X74, X75	E955, E985
Sharp objects	Intentional self-inflicted harm by cutting or sharp object, including cases when its accidental or intentional nature is unknown	X78, X79	E956, E986
Jumping	Intentional self-inflicted harm by jumping from a high place, including cases when its accidental or intentional nature is unknown	X80	E957, E987
Other methods	Intentional self-inflicted harm by non-specified means, vehicles, fire, or vapors; including cases when its accidental or intentional nature is unknown	X76, X77, X81, X82, X83, X84	E958, E988

**Table 2:** Suicides and attempted suicides: frequencies, rates, and ratios *.

Variable		Suicide *N* = 5202 (*n*)	Attempted Suicides *N* = 20,254 (*n*)	Suicide Rates Per 100,000 Inhabitants	Attempted Suicide Rates Per 100,000 Inhabitants	Attempted Suicide to Suicide Rate
Sex	Females	1130	10,821	4.6	43.6	9.58
	Males	4072	9433	17.0	39.3	2.32
Year	2007	708	2542	10.5	37.6	3.59
	2008	822	2939	12.0	42.7	3.58
	2009	749	2922	10.8	42.0	3.90
	2010	685	2784	9.8	39.7	4.06
	2011	651	2861	9.2	40.6	4.39
	2012	781	2925	11.0	41.4	3.75
	2013	806	3281	11.4	46.5	4.07
Age	15–29	509	4087	4.4	35.6	8.03
	30–39	794	5112	8.1	52.1	6.44
	40–44	511	2890	10.8	60.8	5.66
	45–49	494	2396	11.3	55.0	4.85
	50–54	467	1893	12.6	51.0	4.05
	55–59	389	1089	12.6	35.2	2.80
	60–64	372	762	13.2	27.0	2.05
	65–69	317	572	13.1	23.7	1.80
	70–74	382	444	17.9	20.8	1.16
	75–79	400	360	20.7	18.6	0.90
	80-84	332	325	25.3	24.8	0.98
	≥85	235	324	24.5	33.8	1.38

* The chi-squared test for all these associations was significant (*p* < 0.001).

**Table 3:** Adjusted Poisson model for suicides and attempted suicides.

Variable		Poisson Adjusted Model	
		Suicide IRR (95% CI)	Attempted Suicide IRR (95% CI)
Sex	Females	Reference	Reference
	Males	4.02 ^c^ (3.76;4.29)	0.88^c^ (0.86;0.91)
Year	2007	Reference	Reference
	2008	1.13 ^a^ (1.02;1.25)	1.13 ^c^ (1.07;1.19)
	2009	1.02 (0.92;1.12)	1.11 ^c^ (1.05;1.17)
	2010	0.91 (0.82;1.02)	1.05 (0.99;1.1)
	2011	0.86 ^b^ (0.77;0.95)	1.07 ^a^ (1.01;1.13)
	2012	1.01 (0.92;1.12)	1.09 ^b^ (1.03;1.15)
	2013	1.04 (0.94;1.15)	1.22 ^c^ (1.16;1.28)
Age	15–29	Reference	Reference
	30–39	1.84 ^c^ (1.64;2.05)	1.45 ^c^ (1.39;1.52)
	40–44	2.45 ^c^ (2.17;2.77)	1.7 ^c^ (1.61;1.78)
	45–49	2.59 ^c^ (2.29;2.93)	1.53 ^c^ (1.46;1.62)
	50–54	2.89 ^c^ (2.55;3.28)	1.43 ^c^ (1.35;1.51)
	55–59	2.9 ^c^ (2.55;3.31)	0.98 (0.92;1.05)
	60–64	3.08 ^c^ (2.69;3.52)	0.74 ^c^ (0.68;0.8)
	65–69	3.1 ^c^ (2.69;3.56)	0.66 ^c^ (0.6;0.72)
	70–74	4.29 ^c^ (3.76;4.9)	0.58 ^c^ (0.52;0.64)
	75–79	5.12 ^c^ (4.49;5.84)	0.52 ^c^ (0.47;0.58)
	80–84	6.6 ^c^ (5.74;7.58)	0.69 ^c^ (0.61;0.78)
	≥85	7.06 ^c^ (6.04;8.24)	0.93 (0.82;1.05)

^a^*p* < 0.05; ^b^ *p* < 0.01; ^c^ *p* < 0.001; IRR = incidence rate ratio.

**Table 4:** Adjusted Poisson model. Effect of age by sex.

Age (years)	Suicide		Attempted Suicide	
	Females	Males	Females	Males
	IRR (95% CI)		IRR (95% CI)	
15–29	Reference	Reference	Reference	Reference
30–39	2.01 ^c^ (1.55;2.6)	1.80 ^c^ (1.59;2.04)	1.38 ^c^ (1.3;1.47)	1.53 ^c^ (1.44;1.63)
40–44	2.79 ^c^ (2.11;3.69)	2.38 ^c^ (2.08;2.73)	1.64 ^c^ (1.53;1.76)	1.76 ^c^ (1.64;1.89)
45–49	2.88 ^c^ (2.17;3.83)	2.53 ^c^ (2.20;2.90)	1.64 ^c^ (1.52;1.76)	1.42 ^c^ (1.31;1.54)
50–54	3.54 ^c^ (2.67;4.68)	2.74 ^c^ (2.38;3.16)	1.58 ^c^ (1.46;1.7)	1.26 ^c^ (1.15;1.37)
55–59	3.83 ^c^ (2.88;5.1)	2.69 ^c^ (2.32;3.12)	1.01 (0.92;1.11)	0.95 (0.85;1.05)
60–64	4.13 ^c^ (3.1;5.49)	2.83 ^c^ (2.43;3.29)	0.79 ^c^ (0.71;0.88)	0.68 ^c^ (0.6;0.77)
65–69	4.48 ^c^ (3.35;5.98)	2.75 ^c^ (2.34;3.24)	0.72 ^c^ (0.64;0.81)	0.58 ^c^ (0.5;0.67)
70–74	4.75 ^c^ (3.55;6.35)	4.18 ^c^ (3.60;4.86)	0.55 ^c^ (0.48;0.63)	0.61 ^c^ (0.53;0.72)
75–79	4.22 ^c^ (3.11;5.72)	5.43 ^c^ (4.70;6.28)	0.5 ^c^ (0.43;0.59)	0.55 ^c^ (0.46;0.65)
80–84	4.58 ^c^ (3.3;6.34)	7.39 ^c^ (6.34;8.60)	0.56 ^c^ (0.47;0.65)	0.93 (0.79;1.11)
>=85	4.71 ^c^ (3.33;6.65)	8.32 ^c^ (7.00;9.90)	0.81 ^b^ (0.69;0.94)	1.22 (1;1.49)

^a^*p* < 0.05; ^b^ *p* < 0.01; ^c^ *p* < 0.001; IRR = incidence rate ratio.

**Table 5:** Frequency of the method used for suicides and attempted suicides by sex and the results of the adjusted Poisson model for method and sex.

Method	Sex	Suicide *N* = 5202		Attempted Suicide *N* = 6306		Suicide IRR (95% CI)	Attempted Suicide IRR (95% CI)
		*n*	%	*n*	%		
Poisoning by drugs	Males Females	205 168	55.0 45.0	2106 3041	40.9 59.1	Reference 0.84 (0.68, 1.02)	Reference 1.47 (1.40, 1.55)
Poisoning by other means	Males Females	145 85	63.0 37.0	34 18	65.4 34.6	Reference 0.66 (0.50, 0.87)	Reference 0.57 (0.32, 1.02)
Hanging	Males Females	2616 398	86.8 13.2	302 115	72.4 27.6	Reference 0.16 (0.14, 0.17)	Reference 0.39 (0.31, 0.48)
Drowning	Males Females	60 40	60.0 40.0	12 7	63.2 36.8	Reference 0.59 (0.39, 0.90)	Reference 0.51 (0.19, 1.36)
Firearms	Males Females	276 10	96.5 3.5	45 13	77.6 22.4	Reference 0.04 (0.02, 0.79)	Reference 0.34 (0.18, 0.64)
Sharp objects	Males Females	93 21	81.6 18.4	144 89	61.8 38.2	Reference 0.23 (0.14, 0.37)	Reference 0.63 (0.48, 0.82)
Jumping	Males Females	588 373	61.2 38.8	126 68	64.9 35.1	Reference 0.64 (0.57, 0.74)	Reference 0.55 (0.41, 0.74)
Other methods	Males Females	108 40	73.0 27.0	146 140	51.0 49.0	Reference 0.38 (0.26, 0.54)	Reference 0.98 (0.77, 1.23)
Total	Males Females	4091 1135	78.3 21.7	2915 3491	45.5 54.5		

## Author Statement

The authors declare that this manuscript results from an investigation performed by a group of clinical researchers at the Virgen de las Nieves University Hospital of Granada and the University of Granada. These findings on the comparison of factors associated with attempted and completed suicides in the Andalusian population are original and have not previously been published.
